# Supplementary material for: Synthesis, Antimicrobial and Antioxidant Activities of 2-Isoxazoline Derivatives
Source: Molecules. 2020 Sep 18;25(18):4271. doi: 10.3390/molecules25184271 (PMC7570493; doi:10.3390/molecules25184271)
Supplement: Supplementary file 1 [file molecules-25-04271-s001.pdf]

Supplementary Materials for

Synthesis, Antimicrobial and Antioxidant Activities of 2-Isoxazoline Derivatives

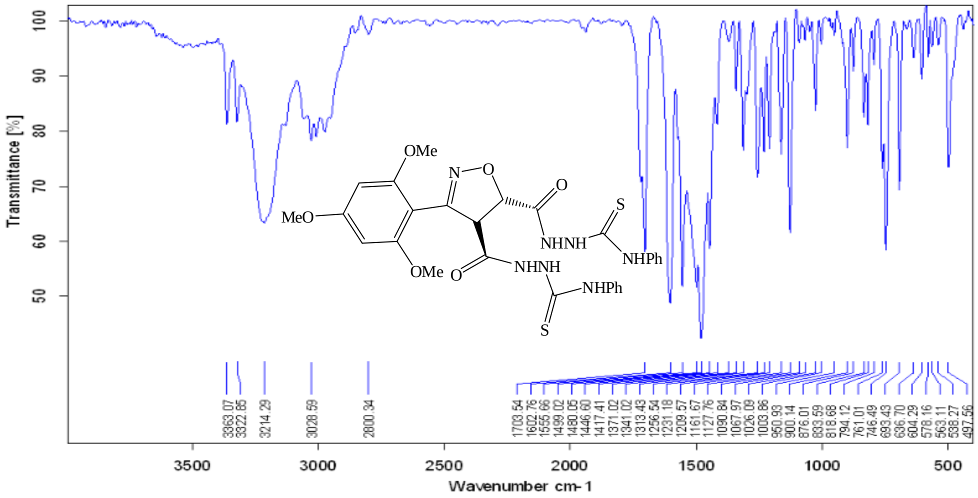

Figure S1: IR (KBr disk) spectrum for compound **9**.

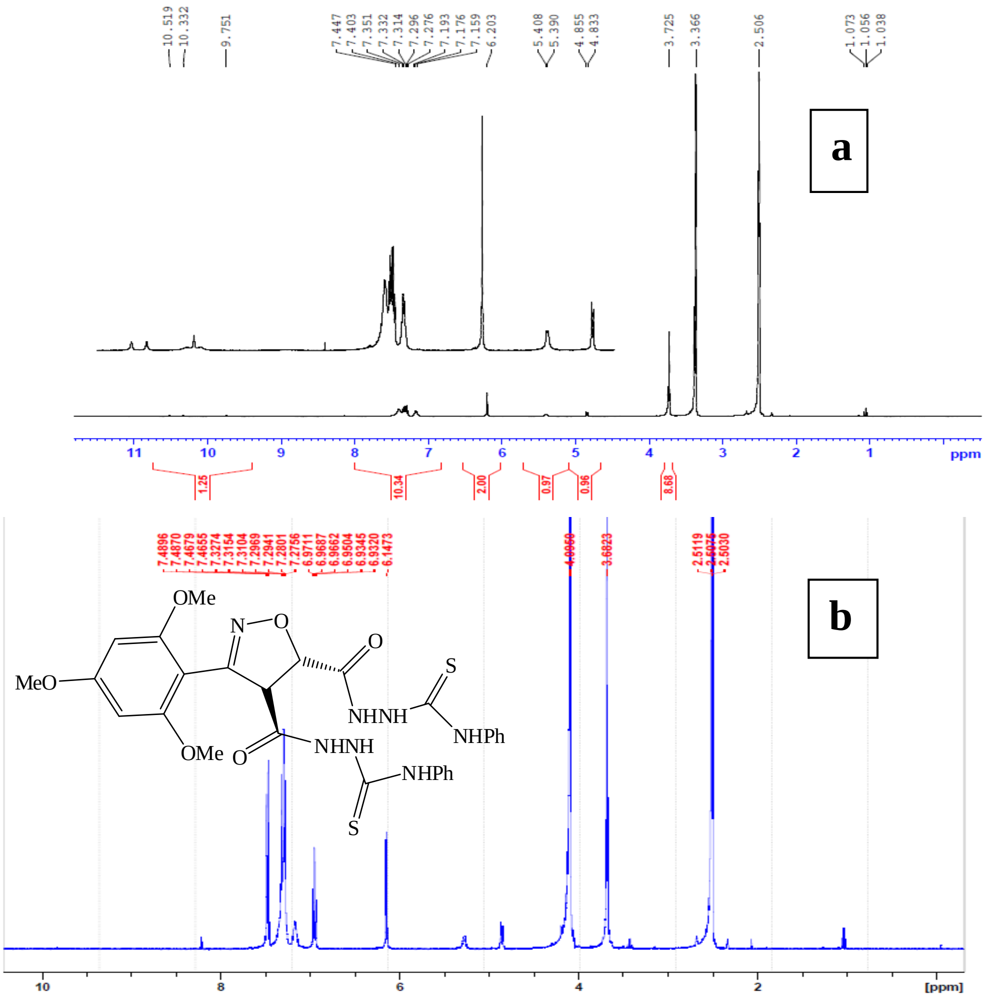

Figure S2: ^{1}H-NMR spectrum of compound **9** in DMSO-d6 Solvent.
a- Without D_2O. b- D_2O added.

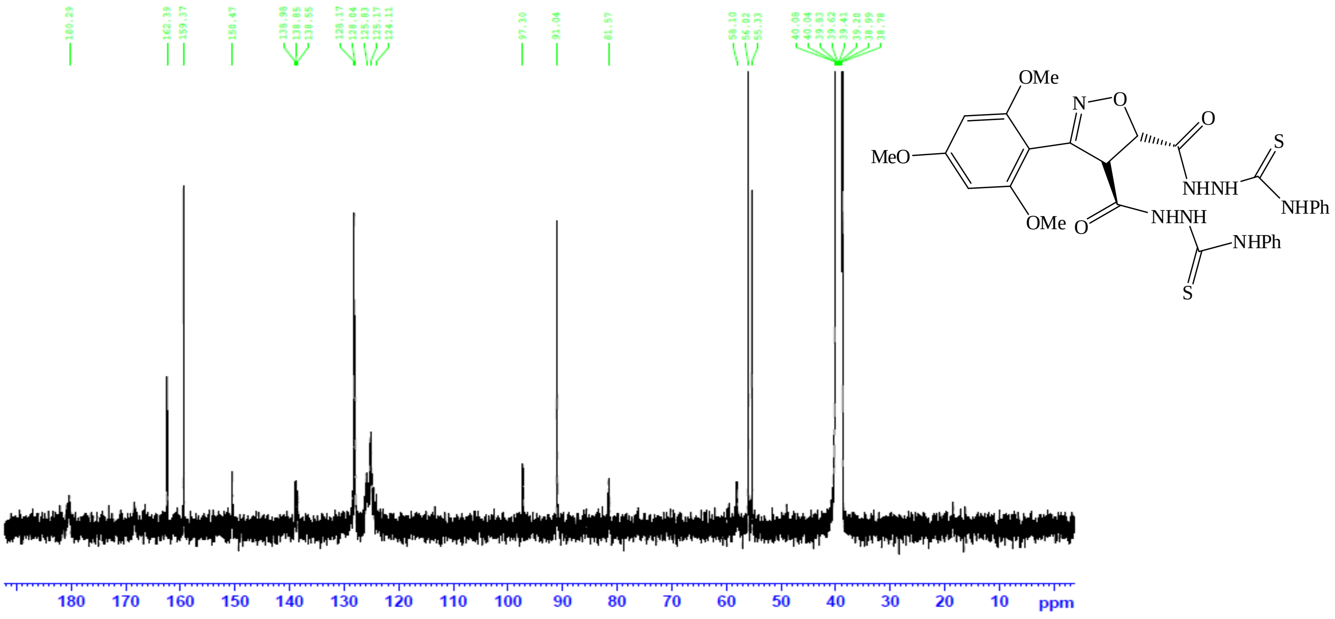

Figure S3: ^{13}C-NMR spectrum of compound **9** in DMSO-d6 Solvent.

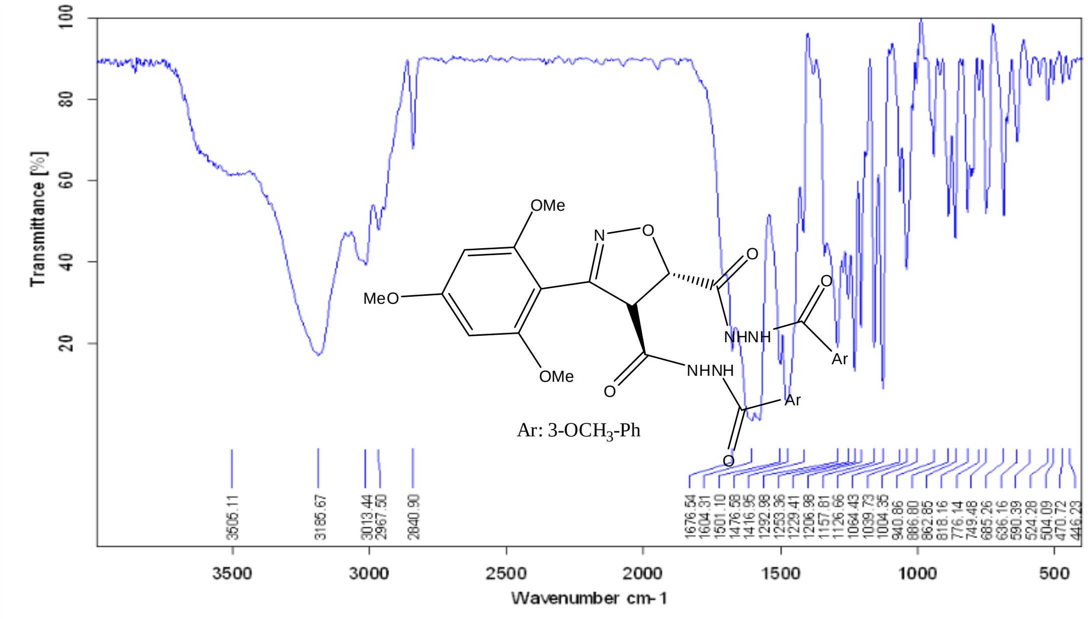

Figure S4: IR (KBr disk) spectrum for compound **10b**.

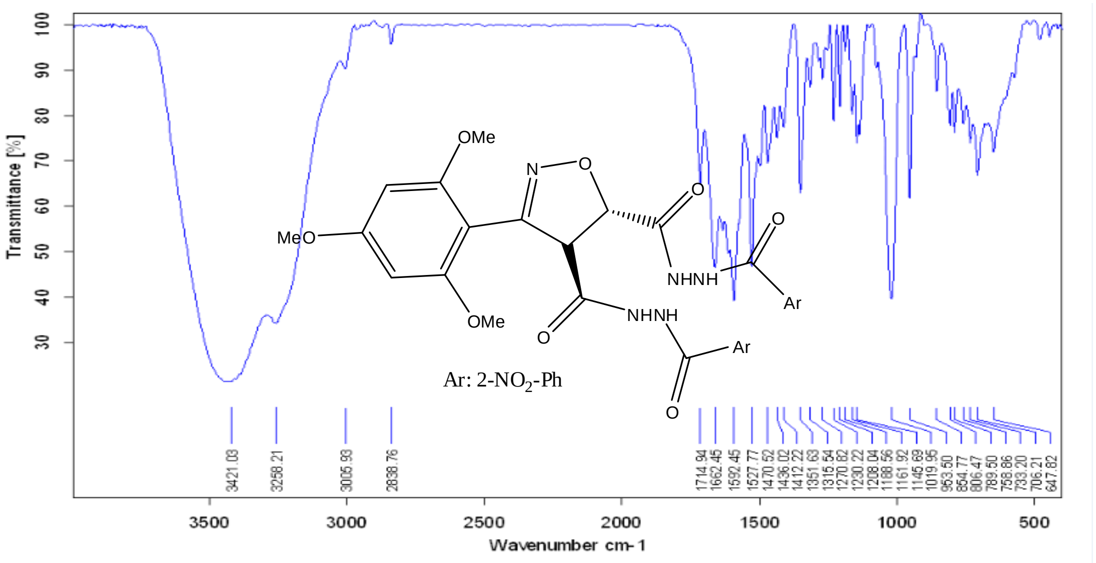

Figure S5: IR (KBr disk) spectrum for compound **10c**.

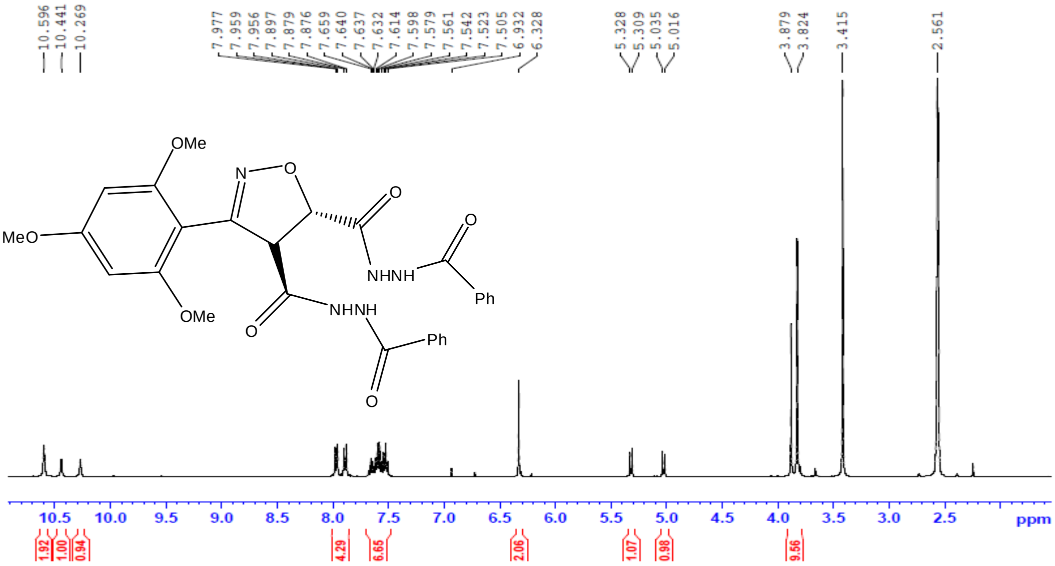

Figure S6: ¹H-NMR spectrum of compound **10a** in DMSO-d6 Solvent.

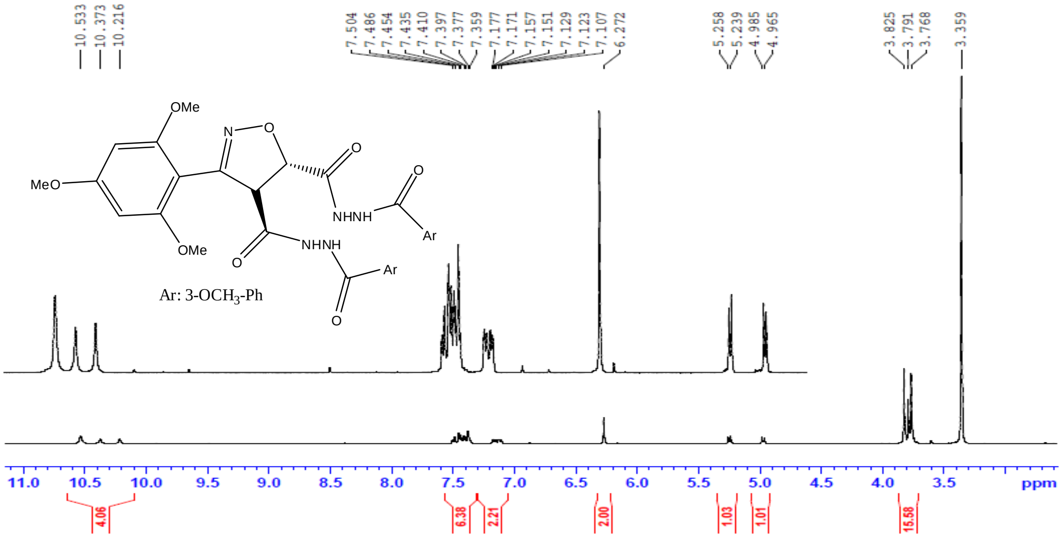

Figure S7: ¹H-NMR spectrum of compound **10b** in DMSO-d6 Solvent.

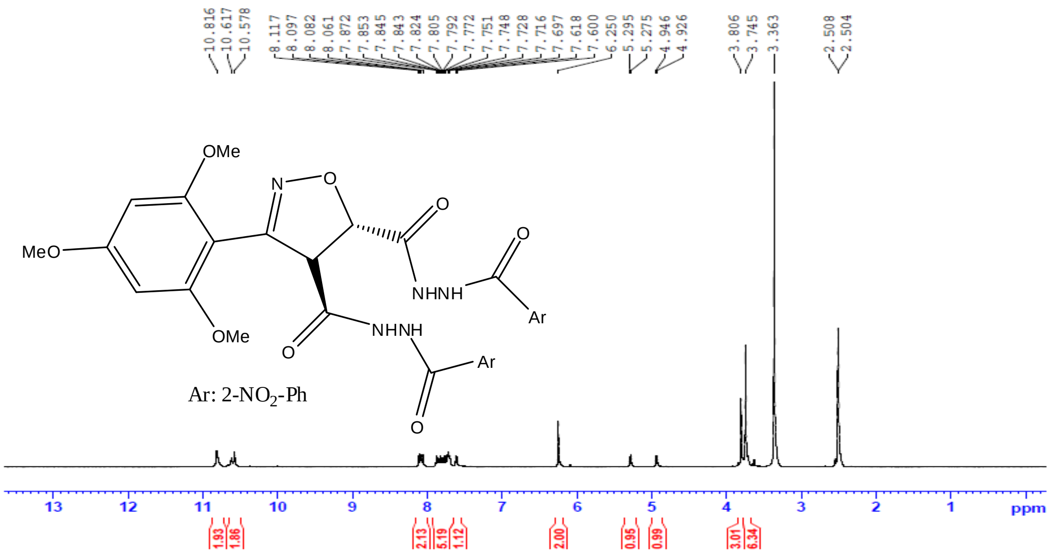

Figure S8: ^{1}H-NMR spectrum of compound **10c** in DMSO-d6 Solvent.

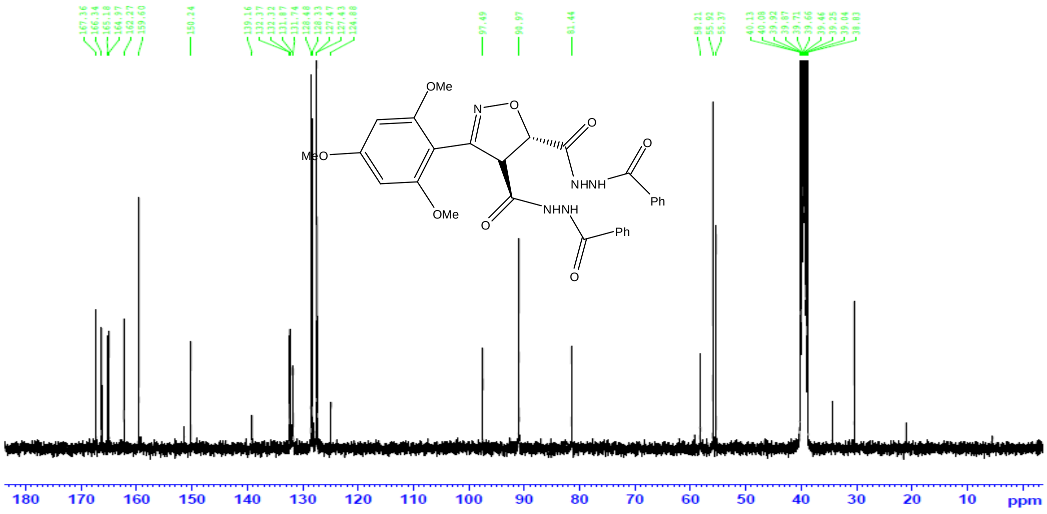

Figure S9: ^{13}C-NMR spectrum of compound **10a** in DMSO-d6 Solvent.

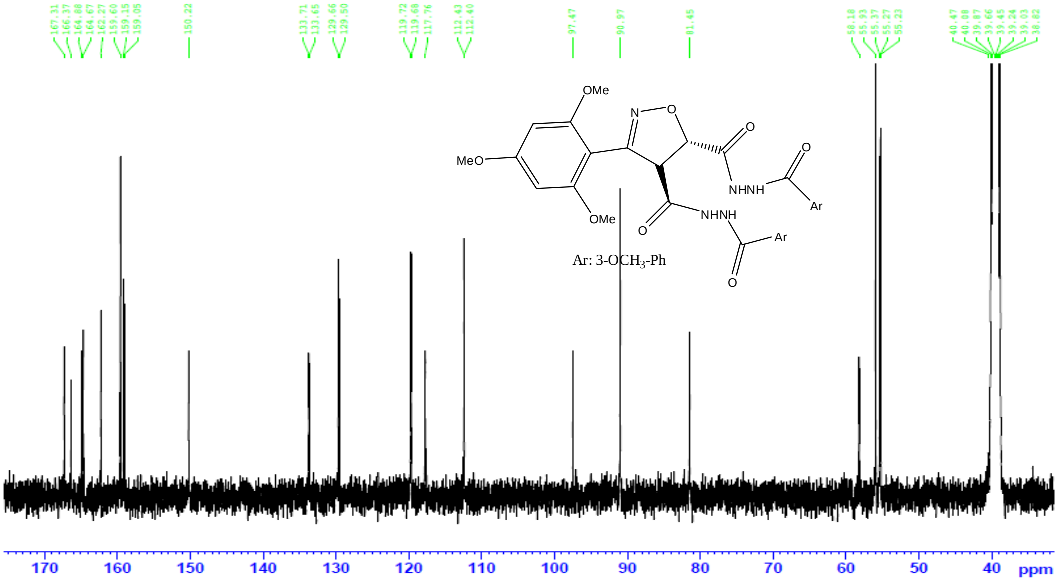

Figure S10: ^{13}C-NMR spectrum of compound **10b** in DMSO-d6 Solvent.

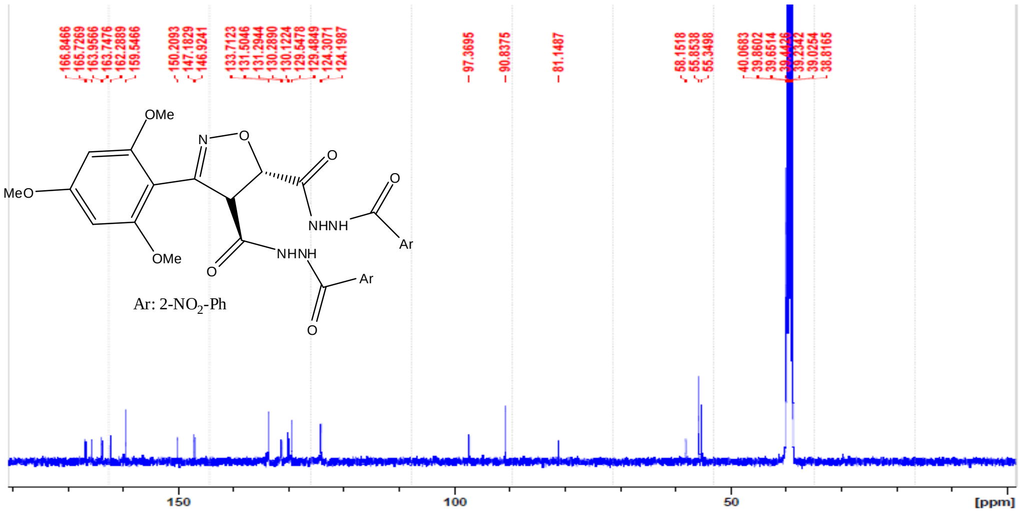

Figure S11: ^{13}C-NMR spectrum of compound **10c** in DMSO-d6 Solvent.

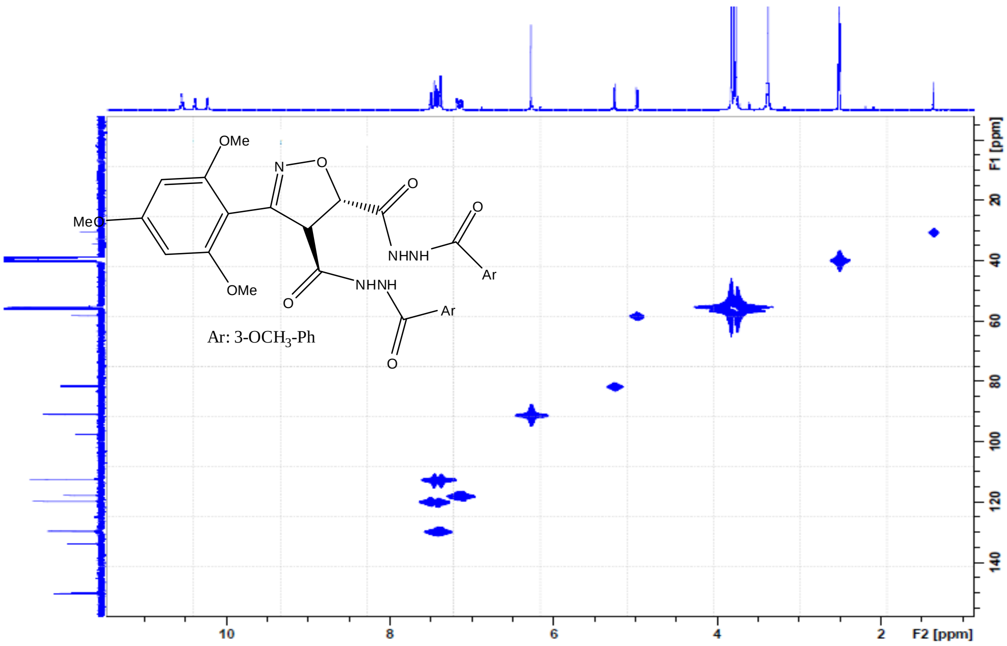

Figure S12: HMQC spectrum of compound **10b** in DMSO-d6 Solvent.

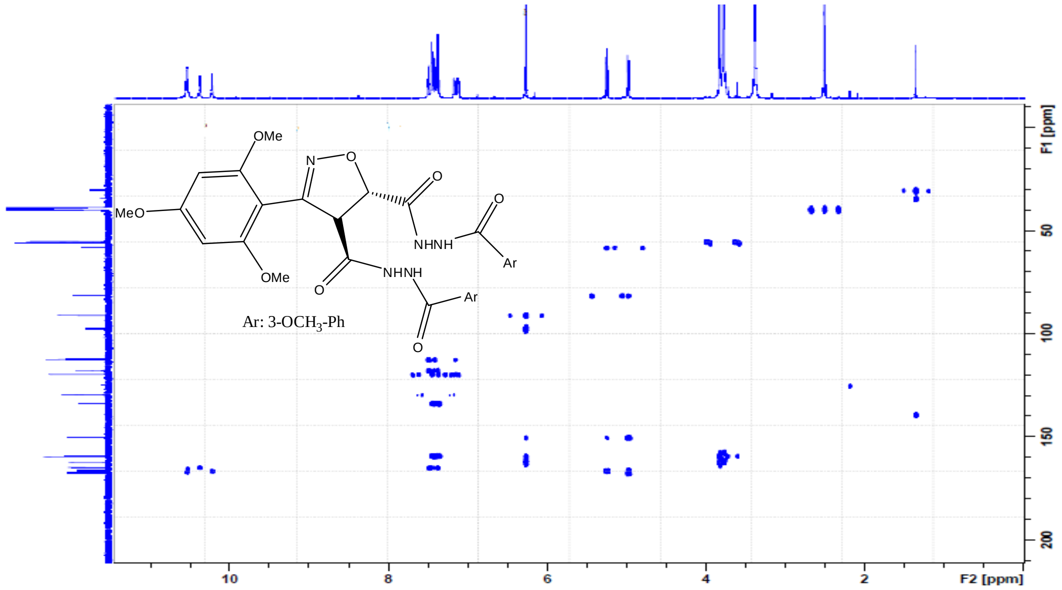

Figure S13: HMBC spectrum of compound **10b** in DMSO-d6 Solvent.
